# Choline-Amino Acid Ionic Liquids as Green Functional Excipients to Enhance Drug Solubility

**DOI:** 10.3390/pharmaceutics10040288

**Published:** 2018-12-19

**Authors:** Rita Caparica, Ana Júlio, André Rolim Baby, Maria Eduarda Machado Araújo, Ana Sofia Fernandes, João Guilherme Costa, Tânia Santos de Almeida

**Affiliations:** 1 CBIOS-Universidade Lusófona’s Research Center for Biosciences & Health Technologies, Campo Grande 376, 1749-024 Lisboa, Portugal; rita.caparica@ulusofona.pt (R.C.); ana.julio@ulusofona.pt (A.J.); ana.fernandes@ulusofona.pt (A.S.F.); jgcosta@ulusofona.pt (J.G.C.); 2 Department of Biomedical Sciences, University of Alcalá, Ctra. Madrid-Barcelona Km. 33.600, Alcalá de Henares, 28871 Madrid, Spain; 3 Department of Pharmacy, School of Pharmaceutical Sciences, University of São Paulo, 580 Prof. Lineu Prestes Av., Bl. 15, São Paulo, SP 05508-900, Brazil; andrerb@usp.br; 4 Centro de Química e Bioquímica, Faculdade de Ciências, Universidade de Lisboa, 1749-016 Lisboa, Portugal; mearaujo@fc.ul.pt; 5 Research Institute for Medicines (iMed.ULisboa), Faculty of Pharmacy, Universidade de Lisboa, Av. Professor Gama Pinto, 1649-003 Lisboa, Portugal

**Keywords:** ionic liquids, solubility/loading enhancement, radical scavenging activity, cell viability, oil-in-water emulsions, stability

## Abstract

The development of effective forms to incorporate poorly soluble drugs into delivery systems remains a problem. Thus, it is important to find alternatives such as finding excipients that increase drug solubility. Ionic liquids (ILs), particularly choline-based ILs, have been studied as solubility enhancers in drug delivery systems. Nonetheless, to acknowledge this property as a functionality, it needs to be proven at non-toxic concentrations. Hence, herein two choline-amino acid ILs were studied as functional excipients by evaluating their influence on the solubility of the poorly water-soluble ferulic acid and rutin, while considering their safety. The solubility of the drugs was always higher in the presence of the ILs than in water. Ionic liquids did not affect the radical scavenging activity of the drugs or the cell viability. Moreover, stable oil-in-water (O/W) emulsions were prepared containing each drug and the ILs, allowing a significantly higher drug loading. Globally, our results suggest that choline-based ILs may act as green functional excipients, since at non-toxic concentrations they considerably improve drug solubility/loading, without influencing the antioxidant activity of the drugs, the cell viability, or the stability of the formulations.

## 1. Introduction

Over the last few years, the pharmaceutical and cosmetic industries have been facing several problems in the development of delivery systems, namely the poor solubility, permeability and stability of certain drugs. In the research and development of new chemical entities (NCEs), the low solubility is a growing and emerging problem [1,2,3]. More than 40% of NCEs developed are practically insoluble in water [4]. Moreover, about 75% of the drugs candidates have low solubility and belong to classes II and IV of the Biopharmaceutical Classification System [1]. Currently, several strategies have been used to overcome this problem, like the encapsulation of actives into nanoparticles [5,6], but there is still much to be done. Hence, it is of the utmost importance to find new ways to overcome this challenge, such as finding eco-friendly excipients that may act as functional ingredients at non-toxic concentrations.

Drug delivery through the skin, namely topical delivery is one of the alternatives routes that has been increasingly explored and which may allow a more effective delivery of drugs to the target site. The topical delivery, which is characterized by the application of drugs for dermatological treatment or cosmetic applications has several advantages. Amongst them, it can be highlighted the avoidance of hepatic first pass metabolism and gastric pH variations, improved patient compliance and acceptance, enhanced drug bioavailability, and direct access to the target site and the fact that it is painless and non-invasive [7,8,9].

Nonetheless, to develop any type of drug delivery system (e.g., oral or topical) it is necessary not only to carefully select and understand the properties of the drug and the excipients, but it is also vital to ensure their safety, individually and combined.

The interest in the use of ionic liquids (ILs) as functional excipients in different formulations has grown in recent years. ILs are organic salts [10,11], with melting points below 100 °C or, in some cases, liquid at room temperature, known as room temperature ionic liquids (RTILs) [12,13,14]. These salts are remarkable chemical compounds, with applications in several areas, due to their numerous valuable properties, such as their ability to dissolve organic, inorganic, and polymeric materials [10,15]. These salts are also non-flammable [12,16,17], have high thermal and chemical stability, have low vapor pressures [10,12,18,19,20,21], and high ionic conductivity [22]. Additionally, their high susceptibility to modifications is probably their most valuable property. Through the deliberate alteration of its ions, it is possible to synthesize ILs with specific physical and chemical properties for a particular application [12,22]. Recently there has been a growing interest in the applicability of ILs in the pharmaceutical field, namely in their utility towards the development of more efficient drug delivery systems, improving the solubility/loading of poorly soluble drugs. Nonetheless, some of these salts are very toxic and thus it is fundamental to know their toxicity and fully understand if these salts still act as functional ingredients at non-toxic concentrations. In fact, a recent study conducted a meta-analysis showing that the amount of publications concerning ILs that consider their toxicity is still very limited, compared to the total number of studies of ILs in literature [23]. Moreover, even though in the last years there has been an increase in the number of studies concerning the toxicity of ionic liquids [24], these results need to be considered when trying to prove the utility of ILs as functional excipients. In fact, to ensure that ILs may have a safe function, this functionality needs to be displayed at concentrations known to be non-cytotoxic and not at higher concentrations.

In this context, choline-based ILs have been shown to be more promising as functional excipients in drug delivery systems containing caffeine, due to their lower toxicity compared to other ILs, such as imidazole-based ILs [14]. To study their impact, at non-toxic concentrations, on the solubility of compounds that have a limited utility, due to their very low solubility, is a rather relevant strategy. It is also important to show the influence of incorporating these salts and higher amounts of drug on the stability of the developed systems.

Ferulic acid and rutin are examples of compounds with pharmaceutical and cosmetic interest, but with very low solubility in water, which limits their delivery and bioavailability. For instance, ferulic acid has been associated with antioxidant, anti-inflammatory, antiallergic, antimicrobial, antiviral, hepatoprotective, anticarcinogenic, antithrombotic, and vasodilatory properties [25,26]. Some studies have also shown that in topical application, ferulic acid in combination with vitamins C and E prevents the onset of erythema, as well as the appearance of wrinkles and hyperpigmentation [25,27].

Rutin is a bioflavonoid known to have antioxidant properties and wide therapeutic actions such as anti-inflammatory, anticancer, antidiabetic, antimicrobial, and neuroprotection effects [28,29]. Studies have also shown that rutin improves skin dermal density, reduces fine wrinkles, and enhances elasticity [30], which shows that this compound may also be relevant for the cosmetic industry.

Thus, both compounds have pharmacological potential, which may be useful in systemic or topical delivery, but their therapeutic value becomes somewhat irrelevant, due to their low solubility.

Consequently, the aim of this work was to study two choline-based ionic liquids, prepared from biomaterials, as green excipients by evaluating their influence on the solubility of the poorly water soluble ferulic acid and rutin, while considering their safety. To achieve this, the influence of the ILs on drug solubility and the impact of the drugs or the systems IL:Drug on the radical scavenging activity and on the cell viability, was assessed. Finally, considering the possible value of incorporating these drugs in topical formulations and to screen the ability of ILs to enhance drug loading, oil-in-water (O/W) emulsions were prepared and their stability was evaluated.

## 2. Materials and Methods

### 2.1. Chemicals

The reagents and solvents used, for ILs synthesis, were choline hydroxide in methanol [Cho][OH]/MeOH 45% (Sigma-Aldrich, Saint Louis, MO, USA), acetonitrile (VWR, Fontenay-sous-Bois, France), methanol (Sigma-Aldrich, Saint Louis, MO, USA), and l-phenylalanine and l-glutamine (PanReact AppliChem, Barcelona, Spain). For cytotoxicity studies, phosphate buffered saline (PBS; 0.01 M, pH 7.4), fetal bovine serum (FBS), trypsin, penicillin–streptomycin (pen/strep) solution, crystal violet (CV), and dimethyl sulfoxide (DMSO) were purchased from Sigma-Aldrich, Saint Louis, MO, USA and Dulbecco’s Modified Eagle’s Medium (DMEM) was provided by Biowest, Nuaillé, France. Ferulic acid and rutin solutions were prepared in DMSO (the final concentration in cell culture was 0.5% (*v/v*) for all assays). Ionic liquid solutions were prepared in sterile water. For the emulsions, Crodafos^®^ CES (cetearyl alcohol (and) dicetyl phosphate (and) ceteth-10 phosphate) was purchased by Mapric, São Paulo, Brazil, isopropyl myristate (Scharlab, Sentmenat, Spain), butylated hydroxytoluene (BHT) (Mapric, São Paulo, Brazil), ethylenediaminetetra-acetic acid disodium dihydrate (Fagron, Barcelona, Spain), propylene glycol (Fragon, Barcelona, Spain), polyethylene glycol 400 (PanReact, Barcelona, Spain), methylparaben and propylparaben (Sigma-Aldrich, Saint Louis, MO, USA), and triethanolamine (José Vaz Pereira, Lisbon, Portugal). For DPPH assay, DPPH (2,2-diphenyl-1-picrylhydrazyl) and methanol were purchased from Sigma-Aldrich, Steinheim, Germany.

Ferulic acid was purchased by Henrifarma, São Paulo, Brazil and rutin by Fragon, São Paulo, Brazil.

### 2.2. Ionic Liquids Synthesis

In this study, two ILs derived from amino acids were prepared according to the literature [14], the 2-hydroxyethyl-trimethylammonium-l-phenylalaninate [Cho][Phe] and the 2-hydroxyethyl-trimethylammonium-l-glutaminate [Cho][Glu]. These ILs were characterized by ^1^H-NMR and ^13^C-NMR spectra, in a Brucker Avance 400^®^ apparatus, Billerica, MA, USA, at 400 MHz, using D_2_O.

### 2.3. Solubility Studies

To perform the solubility studies several saturated solutions of each of the active compounds, ferulic acid or rutin were prepared in water and in several water:IL mixtures. These solutions were then placed on a horizontal shaker (IKA VIBRAX VXR^®^, LTF Labortechnik GmbH & Co, Bodensee, Germany) during 72 h at 25 °C and 32 °C. All solutions were prepared in triplicate and filtrated to remove the excess solute. Then, the samples were analysed by UV-visible spectrophotometry (Evolution^®^ 300, Thermo Scientific, Hertfordshire, England) at the maximum absorption wavelength of each active (313 nm for ferulic acid and 353 nm for rutin).

### 2.4. Cell Culture

Human breast cancer cell line (MDA-MB-231) was obtained from the American Type Culture Collection (ATCC; Manassas, VA, USA) and cultured in DMEM medium supplemented with 10% FBS and 1% pen/strep. Cells were maintained at 37 °C, under a humidifier air atmosphere containing 5% of CO_2_.

### 2.5. Crystal Violet (CV) Staining Assay

Cell viability was evaluated with the CV staining assay. Cells were seeded at a density of 4 × 10^3^ per well in 200 µL culture medium in 96-well plates and incubated for 24 h. Then, cells were incubated with rutin or ferulic acid (0–250 µM) and/or [Cho][Phe] or [Cho][Glu] IL (0–0.2%) for 48 h. The CV staining assay was then carried out, according to a previously described protocol [31,32,33]. Absorbance values for untreated control cells correspond to 100% cell viability. For this assay, two to eight independent experiments were carried out and four replicate cultures at least were used in each independent experiment.

### 2.6. Radical Scavenging Assay with DPPH Radical (DPPH Assay)

The assay was performed according to the method reported in the literature [34]. Briefly, an aliquot (100 µL) of the solutions containing each active, either in water or water:IL (99.8:0.2% *v/v*) mixture, was placed in a stoppered small flask and 3.9 mL of freshly prepared 0.004% methanolic solution of DPPH radical was added. The mixture was shaken and maintained at room temperature (25.0 ± 2.0 °C) in the dark for 1 h [35]. Controls were prepared using the same solvent (water or water:IL mixtures) used to prepare the studied samples. All samples were prepared and analyzed in triplicate. The absorbance values were measured at 517 nm (using a UV-Visible, Evolution^®^ 300, Thermo Scientific, Hertfordshire, England) and the radical scavenging activities (RSA) of the samples were expressed as the percentage inhibition of the DPPH radical and calculated according to the following formula:

RSA(%) = [(*A*_C_ − *A*_S_)/*A*_C_] × 100, (1)

*A*_C_ and *A*_S_ are the absorbance values of the control and the samples, respectively.

### 2.7. Preparation of O/W Emulsions

Emulsions were prepared by incorporating each active, ferulic acid or rutin, with each IL, [Cho][Phe] or [Cho][Glu], into an oil-in-water emulsion according to the qualitative and quantitative (%, *w/w*) composition described in Table 1. For the preparation of the O/W emulsions the compounds were weighted for the oil and aqueous phases. Both phases were heated at 65 °C, in a water bath, and then the aqueous phase was added to the oil phase under stirring and cooled to room temperature.

### 2.8. Accelerated Stability Studies of the O/W Emulsions

The formulations were challenged with a centrifuge test and temperature cycles. In the centrifugation test (n = 3), 5 g of each formulation was heated at 45 °C for 30 minutes and then centrifuged for 30 minutes at 7200 × *g*. The freeze-thaw stability of the O/W emulsions (n = 3) was also performed using temperature cycles. Each cycle was completed by placing the product in the freezer (−10 °C) for 24 h and 45 °C for another 24 h [36,37]. The organoleptic characteristics, pH, and viscosity of all formulations were analyzed at time zero and after 5 temperature cycles.

### 2.9. Real-Time Stability Studies of the O/W Emulsions

The formulations (n = 3) were submitted to heating in the oven (45 °C) or cooling in the freezer (−10 °C), for 3 months. Additionally, to evaluate the behavior of the emulsions under normal storage conditions, the formulations (n = 3) were also stored at room temperature (RT), for 3 months. The organoleptic properties (color, odor, and appearance) and physical–chemical parameters (pH and viscosity) of all formulations were analysed at time zero and after 3 months. The pH was assessed using a pH meter, 827 pH lab, Metrohm^®^, Herisau Switzerland and viscosity using RVDV-I + viscometer, BROOKFIELD^®^, Middleboro, MA, USA.

## 3. Results and Discussion

The two choline-amino acid ionic liquids, [Cho][Glu] and [Cho][Phe], were prepared according to the literature [14]. Both prepared ionic liquids are viscous liquids at room temperature and their spectroscopic data (Figure S1) are in agreement with previously described obtained data [11]. The interest in these ILs relates to the fact that they have been shown to be less toxic and thus more suitable to be incorporated in delivery systems [14].

### 3.1. Solubility Studies

To understand the influence of the ILs on the solubility of the poorly soluble ferulic acid and rutin, solubility studies were performed in water and in different water:IL mixtures. Thus, several percentages of both ILs were studied to assess the influence of the ILs concentration on the solubility of each studied active. Furthermore, even though it is relevant to understand this concentration impact, the main goal of this study was to evaluate if, at non-toxic concentrations (up to 0.2% *v/v*) [14], the studied ILs significantly enhance the solubility of ferulic acid and rutin, to ensure their safe functionality.

**Figure 1:**
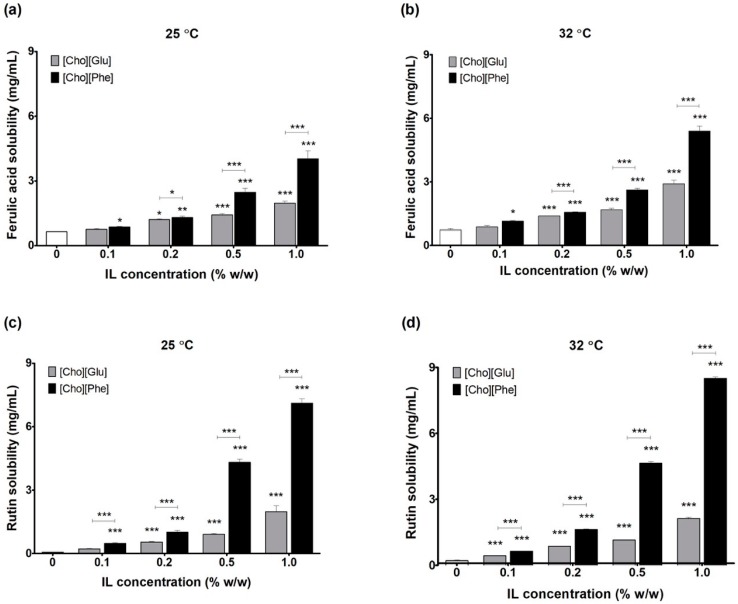
Solubility in water or water:IL mixtures (99.8:0.2%, *w/w*) of ferulic acid at 25 °C (**a**) and 32 °C (**b**) and of rutin at 25 °C (**c**) and 32 °C (**d**). n = 3, mean ± SD and * *p* < 0.05, ** *p* < 0.01, *** *p* < 0.001 (one-way ANOVA, Tukey’s test, relative to water solubility).

The solubility of ferulic acid in water, at 25 °C (Figure 1a), was 0.64 mg/mL, which is in agreement with previously published results of 0.78 mg/mL [38]. For rutin, the solubility, at 25 °C (Figure 1c), was 0.20 mg/mL, which is also in agreement with published results of 0.16 mg/mL [39].

Furthermore, results also show that the solubility of both drugs, at 25 °C and 32 °C, is always higher in the presence of the ILs, than in water (Figure 1). Additionally, when the percentage of the ILs increases, the solubility of the actives also increases, showing that higher amounts of IL allow a higher solubility enhancement of the drugs. Nonetheless, what is more relevant is the fact that even at low concentrations of IL (viz. 0.2%), a considerable enhancement in drug solubility is already attainable. This solubility enhancement is more prominent for rutin (Figure 1c,d). Results also show that amongst the studied ILs [Cho][Phe] proved to be the better solubility promoter, at both studied temperatures, which may reveal a higher affinity between the actives and this IL. This result is in agreement with our previously published results that showed that [Cho][Phe] allowed a higher solubility enhancement of the model active caffeine, when compared to [Cho][Glu] [14].

### 3.2. Impact of ILs Combined with Ferulic Acid and Rutin on Cell Viability

In this study we evaluated the cytotoxicity of the active compounds ferulic acid and rutin, which have been associated in the literature with antitumor properties [40,41,42]. However, there are some controversial reports, in which they did not show cytotoxic effects to some cancer cell lines [43,44]. Thus, since further studies on the influence of these actives in cancer cell models are needed, we used the human breast cancer cell line MDA-MB-231. Furthermore, it is relevant to mention that while previous studies were performed at concentrations up to 100 µM [45,46,47], herein we studied higher concentrations up to 250 µM.

In a previous study from our group [14] we studied the ILs cytotoxicity in HaCaT cells (human keratinocytes), which is a cell line of non-tumor origin. Our results revealed that these ILs have no relevant toxicity up to 0.2%. However, at concentrations higher than 0.2% it was observed a marked decrease in cell viability, indicating that 0.2% should be the upper concentration of ILs that ensures safety for further studies.

**Figure 2:**
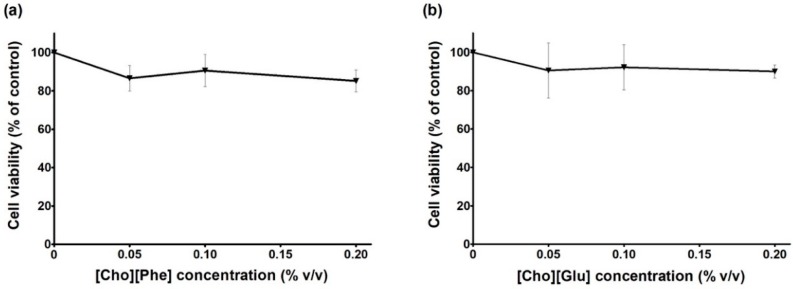
Cell viability of MDA-MB-231 cells exposed to choline-based ILs (0–0.2%, *v/v*), [Cho][Phe] (**a**) and [Cho][Glu] (**b**). The cell viability of ILs-exposed cells (48 h) was evaluated by CV assay. Values represent mean ± SD (n = 6–8) and are expressed as percentages of the non-treated control cells.

In this context, the effect of ILs on cell viability was firstly evaluated in this cell line using the CV staining assay (0–0.2%; 48 h). Exposure to the ILs [Cho][Phe] and [Cho][Glu] did not affect significantly the cell viability, when compared with the control cells (Figure 2). The highest concentration of [Cho][Phe] and [Cho][Glu] evaluated (0.2%) led to a percentage of 85.2 ± 5.6% (Figure 2a) and 90.0 ± 3.5% (Figure 2b) of cell viability, respectively.

**Figure 3:**
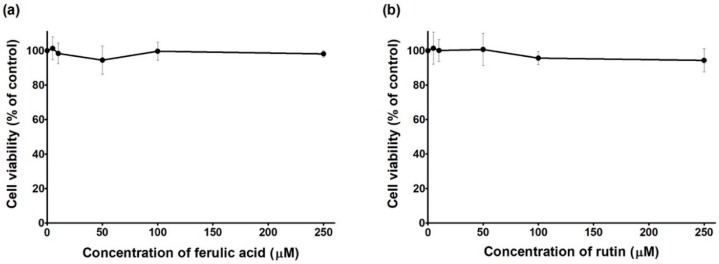
Cell viability of MDA-MB-231 cells exposed to ferulic acid (**a**) and rutin (**b**) for 48 h. The cell viability was evaluated by the CV assay. Values represent mean ± SD (n = 4–5) and are expressed as percentages of the non-treated control cells.

Additionally, it was observed that ferulic acid is not cytotoxic (0–250 µM; 48 h) to this cell line (Figure 3a). These results are in agreement with previous studies performed in other tumor cell lines, in which the treatment with ferulic acid did not show cytotoxic effects [43]. Furthermore, it was also observed that rutin is not cytotoxic (0–250 µM; 48 h) to MDA-MB-231 cells (Figure 3b). This is also in accordance with previous studies, using different tumor cell models [44] or using MDA-MB-231 cells in other experimental conditions [45]. Although the literature is controversial regarding antitumor properties of ferulic acid and rutin [45,46,47], these results could be, at least in part, explained by the highest level of resistance exhibited by MDA-MB-231 cells to several compounds, including chemotherapeutic drugs [48].

**Figure 4:**
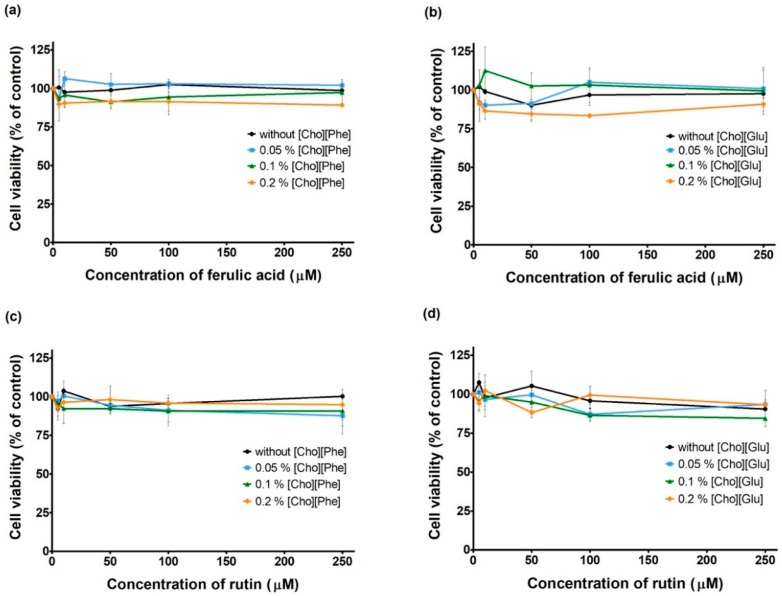
Cell viability of MDA-MB-231 cells exposed to ferulic acid and [Cho][Phe] (**a**) or [Cho][Glu] (**b**) and rutin and [Cho][Phe] (**c**) or [Glu] (**d**). The cell viability of active:IL mixture-exposed cells (48 h) was evaluated by CV assay. Values represent mean ± SD (n = 2–3) and are expressed as percentages of the non-treated control cells.

It is important to highlight that it is crucial to evaluate not only the individual effects of the actives’ treatments (Figure 3) but also the co-treatment with the actives in the presence of the ILs on cell viability. To the best of our knowledge, this was considered herein for the first time and our results show that the treatment of each drug in the presence of either studied ILs (0–0.2%) also showed no significant impact on cell viability (Figure 4).

Globally, these results reveal that the presence of both studied ILs does not markedly influence the cell viability results observed upon treatment with each drug. This is a relevant finding when considering the use of ILs as green functional excipients in drug delivery systems. 

### 3.3. Antioxidant Activity by DPPH Assay

Ferulic acid and rutin are well known as antioxidants compounds [29,49] so it is also important to understand if this activity changes in the presence of the ILs under study. For this purpose, the antioxidant activity (AA) of each active was determined using the DPPH assay. For each drug the RSA was determined, at the concentration corresponding to the maximum solubility of each drug in water at 25 °C (MS_w_), 0.64 mg/mL for ferulic acid and 0.2 mg/mL for rutin. This assessment was made in water and water:IL mixtures, to evaluate the influence of each IL, [Cho][Phe] or [Cho][Glu], on the AA of each active. The percentage of IL used was 0.2% (*v/v*) since it is known to be the highest concentration where cell viability is maintained [14]. Furthermore, the RSA was also determined, at the maximum solubility of each drug, in the presence of water:[Cho][Phe], (MS_w:[Cho][Phe]_), and water:[Cho][Glu], (MS_w:[Cho][Glu]_), (99.8:0.2% *v/v*). The solubility in the presence of [Cho][Phe] was 1.30 mg/mL for ferulic acid and 1.15 mg/mL for rutin and, in the presence of [Cho][Glu] was 1.20 mg/mL for ferulic acid and 0.68 mg/mL for rutin. This was done to ensure that the higher loading achieved, in the presence of the ILs, is translated in a higher RSA of the more concentrated solutions.

For ferulic acid at the MS_w_ (0.64 mg/mL), RSA values in water (78.5 ± 2.6%) and in water:[Cho][Phe] and water:[Cho][Glu] have no significant differences (78.1 ± 4.3% and 79.9 ± 1.1%, respectively), which shows that the presence of the ILs do not influence the RSA of this drug (Table 2). Furthermore, when using ferulic acid at the MS_w:[Cho][Phe]_ and MS_w:[Cho][Glu]_ (1.30 mg/mL and 1.20 mg/mL, respectively), there was an enhancement of about 10% in RSA (to 89.9% and 88.8%, respectively), which agrees with a higher drug content.

For rutin, a similar pattern was observed (Table 2). Once again, at MS_w_ (0.2 mg/mL) the RSA values in water (20.5 ± 0.3%) and in the water:IL mixtures, water:[Cho][Phe] and water:[Cho][Glu], showed no significant differences (20.4 ± 0.7% and 19.5 ± 0.2%, respectively; *p* < 0.05). Additionally, the RSA in water:IL mixtures (99.8:0.2% *v/v*) at the MS_w:[Cho][Phe]_ (1.15 mg/mL) and the MS_w:[Cho][Glu]_ (0.68 mg/mL) was once again higher (RSA of about 90% and 50% of RSA, respectively). This increase was greater for rutin, since the enhancement in drug solubility is also more marked for this active. In fact, when compared to water alone, this enhancement reflects a 3-fold increase in drug solubility in the water:[Cho][Glu] mixture and a 6-fold increase, in the water:[Cho][Phe] mixture (Figure 1). This allows a considerably higher rutin content and, as expected, is reflected in a greater RSA.

Once again, results show that the presence of ILs does not influence the drug activity but allows a higher drug solubility/content that leads to a higher activity of the more concentrated solutions. 

Thus, present results indicate that the use of [Cho][Phe] or [Cho][Glu] combined with ferulic acid or rutin, may be a successful tactic to overcome the drug solubility challenges and to potentially make full use of the therapeutic value of these drugs in different delivery systems. Furthermore, topical formulations containing ILs may be an important way to deliver these poorly soluble drugs to a specific site. To ensure this, it is necessary to show that the presence of ILs, at non-toxic concentrations, allow not only a higher solubility, but also a higher drug loading, in the developed systems. To evaluate this possibility, O/W emulsions containing ferulic acid or rutin and each IL were prepared to evaluate if this strategy enables a higher drug loading.

### 3.4. Drug Incorporation in O/W Emulsions and Stability Studies

Ferulic acid and rutin were incorporated into O/W emulsions in the presence of both ILs, [Cho][Phe] or [Cho][Glu], at a percentage where cell viability is maintained (0.2% *v/v*) [14]. Considering this percentage is crucial, since to show that these ILs truly act as functional ingredients, this functionality needs to be proven at non-toxic concentrations. Both drugs were successfully incorporated in the formulations at the maximum concentration the mixtures water:IL (99.8:0.2% *w/w*) allows to dissolve. This was accomplished accordingly to the solubility results obtained herein (1.20 mg/mL for ferulic acid and 0.68 mg/mL for rutin, in water:[Cho][Glu] and 1.30 mg/mL for ferulic acid and 1.15 mg/mL for rutin, in water:[Cho][Phe]). Formulations containing either ferulic acid or rutin, in the absence of both ILs, were not possible to prepare due to the low solubility of both drugs.

A recent study referred that the thermal decomposition of choline-amino acid ILs occurs at temperatures significantly higher to those used herein for the preparation of the O/W emulsions, which is also relevant to consider when incorporating these amino acid ILs in this type of formulations [21].

Moreover, accelerated stability studies (centrifuge test and temperature cycles) were performed, and all formulations proved to be stable, regarding pH value, viscosity, and organoleptic properties (Table 3). These initial stability tests are the first and fundamental screening to be performed, since many formulations with stability issues, show these problems immediately after submitting them to these preliminary extreme conditions. Thus, these results are a good indicative of the stability of the formulations in the presence of ILs. Nonetheless, stability studies were also performed after 3 months at −10 °C, at 45 °C and at RT. After this period no observable differences were detected, in terms of appearance, color, and odor (organoleptic properties) of the formulations. Also, no considerable changes were detected in the pH and viscosity values (Table 3), and the controls and the formulations showed similar variations. More importantly, no creaming or phase separation was observed during the 3 months indicating that the formulations are stable over the studied time.

These results suggest that the presence of ILs, at non-toxic concentrations, allows a considerably higher drug loading, particularly for rutin, while maintaining the stability of the prepared formulations. This shows that the studied ILs may act as green functional excipients.

## 4. Conclusions

Herein, two choline-based ILs, prepared from biomaterials, [Cho][Phe] and [Cho][Glu] were studied as green excipients to enhance drug solubility and loading of the poorly soluble ferulic acid and rutin, while considering both safety and efficacy.

Results showed that both studied ILs enhance the solubility of the poorly water soluble ferulic acid and rutin, both at 25 °C and 32 °C, although [Cho][Phe] allows a higher enhancement in drug solubility. Moreover, this enhancement increases with the concentration of the ILs. Results from cytotoxicity studies showed that ferulic acid and rutin were not cytotoxic to the studied cell line under our experimental conditions. Additionally, when considering the system IL-drug, results also showed that the presence of the ILs at non-toxic concentrations do not significantly influence the viability of drugs-treated cells.

Concerning the antioxidant activity of both drugs, RSA results show that the presence of the ILs has no influence on the AA of both studied drugs. Nonetheless, by allowing a higher drug solubility/content it enables a higher activity of the more concentrated solutions. Thus, results from cytotoxicity and RSA studies show that the ILs under study do not influence the pharmacological potential of both drugs.

Since at concentrations of ILs where cell viability is maintained (0.2% *v/v*) there is already a significant enhancement in drug solubility, particularly for rutin where a 6-fold enhancement is observed in the presence of [Cho][Phe], the incorporation of each drug in O/W emulsions was also studied, to evaluate if a higher drug loading was also possible when using the ILs as excipients.

The developed O/W emulsions contained each IL and ferulic acid or rutin at the maximum concentration the mixture water:IL (99.8:0.2% *w/w*) allowed to dissolve each drug. All prepared formulations were stable after the performed stability studies, thus showing that ILs did not negatively affect the integrity of the emulsions, while allowing a higher drug loading. 

Thus, this study shows that the choline-based ILs may act as green excipients, since their presence, at non-toxic concentrations, enhanced drug solubility and loading, while ensuring the stability and safety of the delivery systems.

Hence, this study shows the potential of using these ILs to increase the applicability of poorly soluble drugs, with pharmaceutical interest, that otherwise would have a limited function.

## Figures and Tables

**Table 1:** Qualitative and quantitative composition (%, *w/w*) for emulsions: with and without ferulic acid and/or each ionic liquid (IL); with and without rutin and/or each IL. O/W: oil-in-water.

Drug	Composition	O/W Emulsions		
		Control	[Cho][Phe]	[Cho][Glu]
Ferulic Acid	Crodafos^®^ CES	4 or 6	4	6
	Isopropyl myristate	2	2	2
	Butylated hydroxytoluene (BHT)	0.1	0.1	0.1
	Ethylenediaminetetraacetic acid disodium dihydrate (EDTA Na_2_)	0.1	0.1	0.1
	Propylene glycol (PG)	5	5	5
	Polyethylene glycol (PEG 400)	5	5	5
	Parabens solution	1	1	1
	Ferulic acid	–	0.13	0.12
	IL	–	0.2	0.2
	Triethanolamine	q.s. to pH = 5 q.s. to 100		
	Deionized water			
Rutin	Crodafos^®^ CES	4 or 6	4	6
	Isopropyl myristate	2	2	2
	Butylated hydroxytoluene (BHT)	0.1	0.1	0.1
	Ethylenediaminetetraacetic acid disodium dihydrate (EDTA Na_2_)	0.1	0.1	0.1
	Propylene glycol (PG)	5	5	5
	Polyethylene glycol (PEG 400)	5	5	5
	Parabens solution	1	1	1
	Rutin	–	0.115	0.068
	IL	–	0.2	0.2
	Triethanolamine	q.s. to pH = 5 q.s. to 100		
	Deionized water			

**Table 2:** Radical scavenging activity (RSA) of ferulic acid and rutin, in water and in water:IL (99.8:0.2% *v/v*) mixtures, expressed as the percentage inhibition of the DPPH radical.

	Solvent	Drug Concentration (mg/mL)	RSA (%)
Ferulic Acid	Water	0.64	78.5 ± 2.6 ^a^
	Water:[Cho][Phe]	0.64	78.1 ± 4.3 ^a^
		1.30	89.9 ± 1.8 ^b^
	Water:[Cho][Glu]	0.64	79.9 ± 1.1 ^a^
		1.20	88.8 ± 0.5 ^b^
Rutin	Water	0.20	20.5 ± 1.3 ^a^
	Water:[Cho][Phe]	0.20	20.4 ± 0.7 ^a^
		1.15	93.6 ± 0.3 ^b^
	Water:[Cho][Glu]	0.20	19.5 ± 0.2 ^a^
		0.68	56.2 ± 0.4 ^c^

Each uncertainty represents the standard deviation of the mean of three experiments (each with three replicates), mean ± SD. Different letters (a, b, c) in RSA values are indicative of significant statistical differences between the values, within each active (ferulic acid or rutin), with at least *p* < 0.05 (ANOVA, Tukey’s test).

**Table 3:** Stability studies of the O/W emulsions (n = 3): pH and viscosity (visc.) values at time 0, after 5 temperature cycles and after 3 months (at −10 °C, 45 °C, and at RT).

Formulation	Crodafos^®^ CES (%)			Accelerated Stability Studies		Stability Studies (after 3 Months)				RT Stability Studies (after 3 Months)	
		Time Zero		After 5 cycles		−10 °C		45 °C		RT	
		pH	Visc. (mPas)	pH	Visc. (mPas)	pH	Visc. (mPas)	pH	Visc. (mPas)	pH	Visc. (mPas)
Control	4	4.99	10,260	5.00	11,503	4.8	12,400	4.9	12,300	4.8	12,350
FA/[Cho][Phe]		5.00	12,500	4.68	14,700	4.5	15,530	4.5	15,600	4.5	15,400
Rut/[Cho][Phe]		5.02	14,300	4.35	19,630	4.7	20,000	4.8	19,980	4.8	20,060
Control	6	4.99	17,130	5.00	19,100	4.7	20,200	4.8	20,140	4.8	20,100
FA/[Cho][Glu]		5.05	19,600	4.50	21,450	4.45	22,600	4.6	22,660	4.5	22,500
Rut/[Cho][Glu]		4.99	19,000	4.50	22,800	4.5	23,130	4.6	23,000	4.6	23,060

## Supplementary Materials

The following are available online at http://www.mdpi.com/1999-4923/10/4/288/s1, Figure S1: NMR data and structure of the choline-based ionic liquids: a) 1H-NMR of [Cho][Phe]; b) 13C-NMR of [Cho][Phe]; c) 1H-NMR of [Cho][Glu]; d) 13C-NMR of [Cho][Glu].
