# Supplementary material for: Choline-Amino Acid Ionic Liquids as Green Functional Excipients to Enhance Drug Solubility
Source: Pharmaceutics. 2018 Dec 19;10(4):288. doi: 10.3390/pharmaceutics10040288 (PMC6321021; doi:10.3390/pharmaceutics10040288)
Supplement: Supplementary file 1 [file pharmaceutics-10-00288-s001.pdf]

pharmaceutics

MDPI

Supplementary Materials: Choline-Amino Acid Ionic Liquids as Green Functional Excipients to Enhance Drug Solubility

Rita Caparica, Ana Júlio, André Rolim Baby, Maria Eduarda Machado Araújo, Ana Sofia Fernandes, João Guilherme Costa and Tânia Santos de Almeida

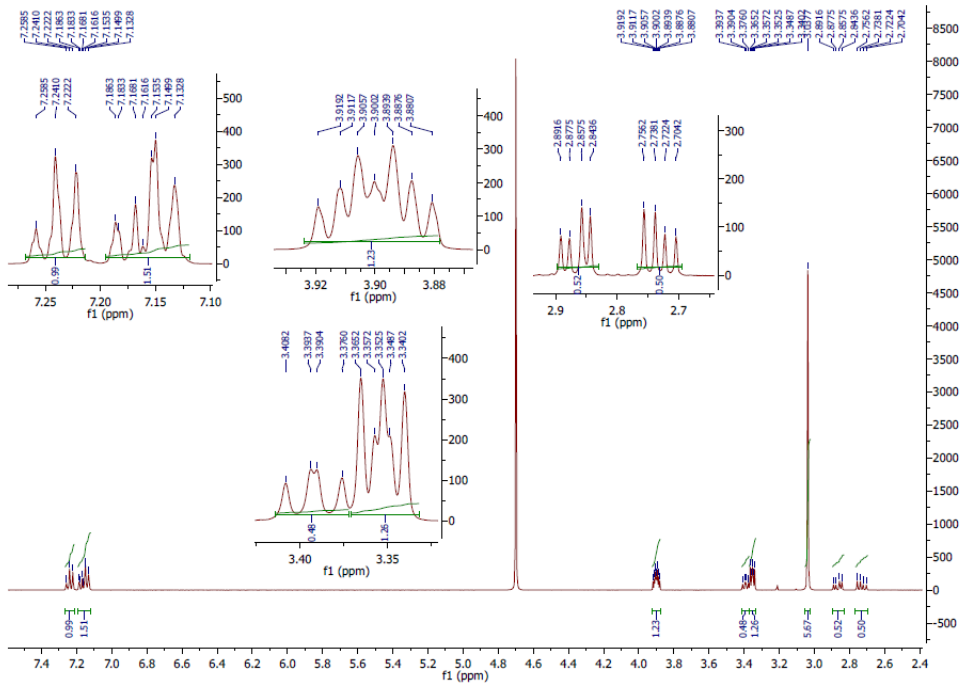

a). **^{1}HNMR (D_2O)** (δ ppm, mult, $J_{H\text{-}H/Hz}$) [Cho][Phe]: 3.04 (s, CH_3, **N-CH_3**), 3.35 (t, 5.1, CH_2, **H-2,**), 3.90 (m, CH_2, **H-3**), 3.39 (dd, J_1= 5.8, J_2=4.76, CH, **H-2′**), 2.87 (dd, J_1= 5.64, J_2=5.56, CH_2, **H-3′**), 2.73 (dd, J_1= 7.24, J_2=7.28, CH_2, **H-4′**), 7.16 (m, $CH_{aromatic\ rimg}$, **H-2′′,3′′,5′′,6′′**), 7.24 (t,7.5, $CH_{aromatic\ ring}$, **H-4′′**).

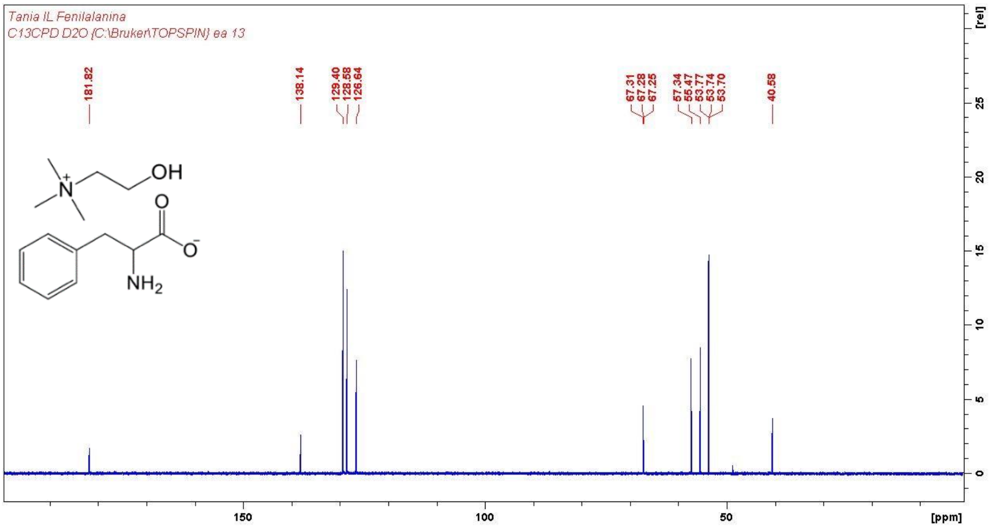

b). **^{13}C NMR (D_2O)** [Cho] [Phe] (δ ppm): 181,8 (COO^-, C1′), 138,14 (C, C1′′), 129,40 (CH, C2′′, C6′′), 128,58 (CH, C3′′, C5′′), 126,64 (CH, C4′′), 67,28 (CH2, C2), 57.34 (CH, C2′), 55.47 (CH_2, C3), 53.77, 53.74, 53.70 (CH_3, C1), 40.58 (CH_2, C3′).

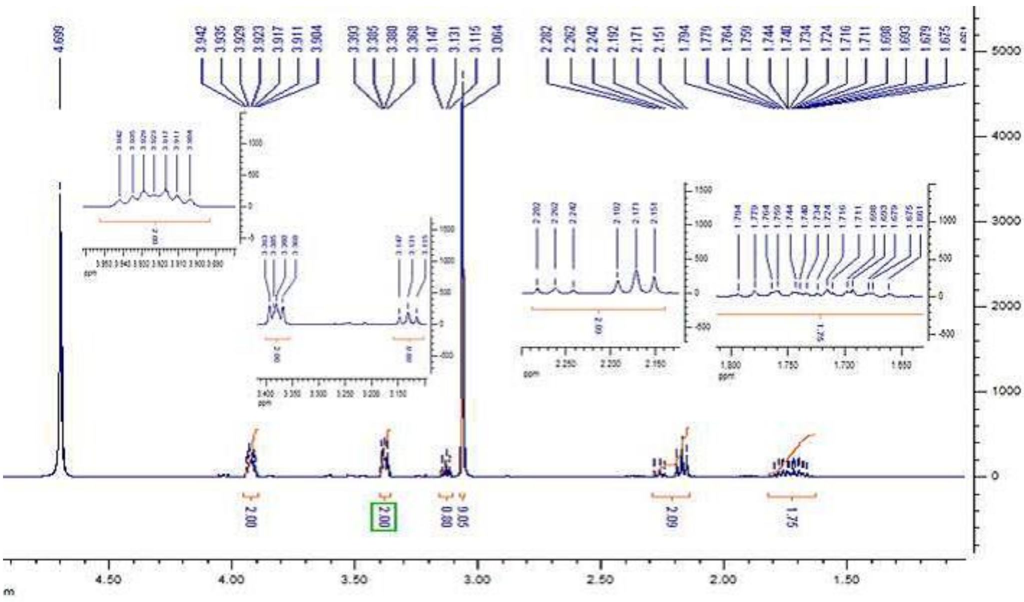

c). ^{1}HNMR (D_2O)[Cho][Glu] (δ ppm, mult, $J_{H\text{-}H/Hz}$): 3.04 (s, CH_3,**N-CH_3),** 3.38 (m, CH_2, **H-2**), 3.92 (m, CH_2, **H-3**), 3.13 (t, 6.3, CH, **H-2′**),1.73 (m, CH_2,**H-3′**), 2.17, 2.26(dt, J=8.17,CH_2, **H-4′**).

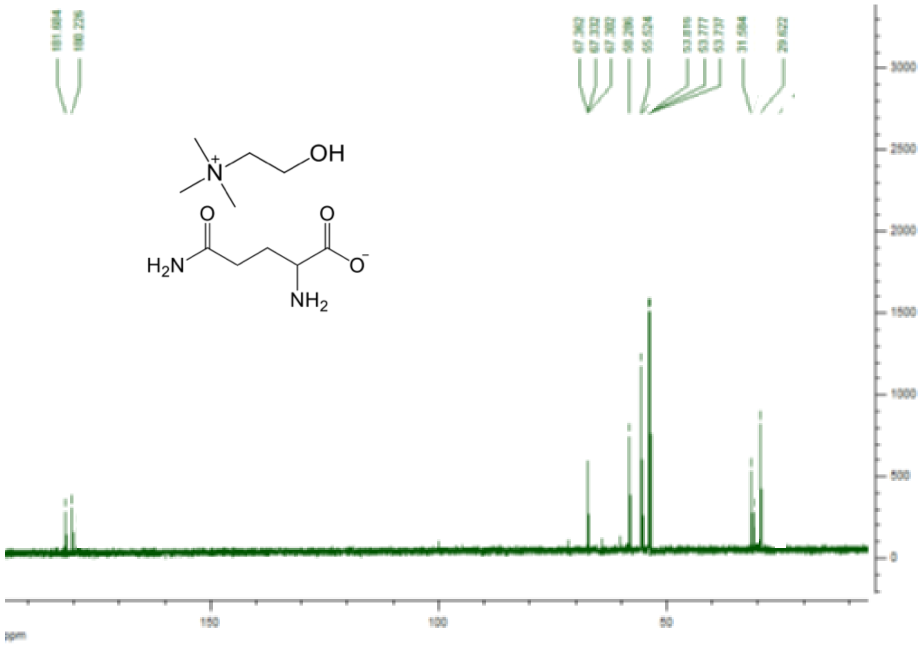

d). ^{13}C NMR (D_2O) [Cho][Glu] (δ ppm): 181.6 ($CONH_2$, C5′), 180.2 (COO^-, C1′), 67.33 (CH_2, C2), 58.29 (CH, C2′),55.52 (CH_2, C3), 53.82, 53.78, 53.74 (CH_3, C1), 31.58 (CH_2, C3′), 29.62 (CH_2, C4′).

Figure S1: NMR data and structure of the choline-based ionic liquids: **a)** ^{1}H-NMR of [Cho][Phe]; **b)** ^{13}C-NMR of [Cho][Phe]; **c)** ^{1}H-NMR of [Cho][Glu]; **d)** ^{13}C-NMR of [Cho][Glu].
